# Medical Institute of Louisville

**Published:** 1844-10

**Authors:** 


### MEDICAL INSTITUTE OF LOUISVILLE.

At the Medical Institute, every morning, may be seen quite a gathering of students, and the number is constantly increasing. A large class will, no doubt, be in attendance by the first of November. The readiness with which students avail themselves of this preliminary course of lectures, augurs well. We hope it will become the settled policy of the school thus to extend the term of instruction.

